# Tailoring the electrochemical properties of 2D-hBN *via* physical linear defects: physicochemical, computational and electrochemical characterisation†

**DOI:** 10.1039/c9na00530g

**Published:** 2019-11-21

**Authors:** Alejandro García-Miranda Ferrari, Dale A. C. Brownson, Ahmed S. Abo Dena, Christopher W. Foster, Samuel J. Rowley-Neale, Craig E. Banks

**Affiliations:** Faculty of Science and Engineering, Manchester Metropolitan University Chester Street Manchester M1 5GD UK c.banks@mmu.ac.uk +44 (0)1612471196; Manchester Fuel Cell Innovation Centre, Manchester Metropolitan University Chester Street Manchester M1 5GD UK; Faculty of Oral and Dental Medicine, Future University in Egypt (FUE) New Cairo Egypt; National Organization for Drug Control and Research (NODCAR) P.O. Box 29 Giza Egypt

## Abstract

Monolayer hexagonal-boron nitride films (2D-hBN) are typically reported within the literature to be electrochemically inactive due to their considerable band gap (*ca.* 5.2–5.8 eV). It is demonstrated herein that introducing physical linear defects (PLDs) upon the basal plane surface of 2D-hBN gives rise to electrochemically useful signatures. The reason for this transformation from insulator to semiconductor (inferred from physicochemical and computational characterisation) is likely due to full hydrogenation and oxygen passivation of the boron and/or nitrogen at edge sites. This results in a decrease in the band gap (from *ca.* 6.11 to 2.36/2.84 eV; theoretical calculated values, for the fully hydrogenated oxygen passivation at the N or B respectively). The 2D-hBN films are shown to be tailored through the introduction of PLDs, with the electrochemical behaviour dependent upon the surface coverage of edge plane-sites/defects, which is correlated with electrochemical performance towards redox probes (hexaammineruthenium(iii) chloride and Fe^2+/3+^) and the hydrogen evolution reaction. This manuscript de-convolutes, for the first time, the fundamental electron transfer properties of 2D-hBN, demonstrating that through implementation of PLDs, one can beneficially tailor the electrochemical properties of this nanomaterial.

## Abstract 2

Electrochemically inert CVD grown 2D-hBN is transformed to give rise to electrochemically useful signatures through the introduction of physical linear defects.

## Graphical abstract

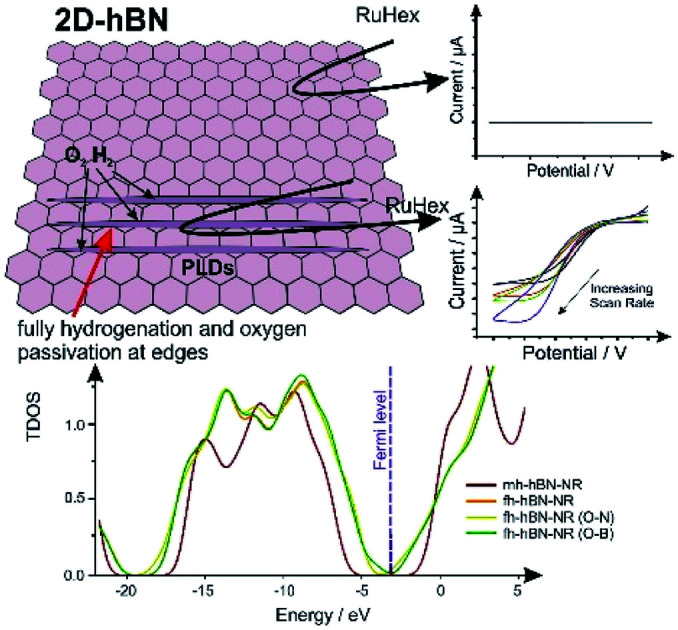

## 1. Introduction

A family of 2D materials such as 2D-hBN, MoSe_2_, MoS_2_, WSe_2_, antimonene and phosphorene^1,2^ have recently been isolated and utilised within an array of electrochemical applications, ranging from sensing to energy storage and generation.^1,3^ Boron nitride (BN) is a structural analogue of graphite, in which an equal number of boron and nitrogen atoms form a honeycomb lattice structure^4^ of sp^2^ bonded layers.^5^ This structure is not found naturally and was first synthesised by Balmain^6^ in 1842. Hexagonal boron nitride (2D-hBN) is a structural analogue of graphene and has a high thermal conductivity and robustness to oxidation,^7^ which historically has allowed it to be used as a lubricant.^8^ Few layer 2D-hBN has also been utilised to improve the mechanical properties of composites, even at low percentages^9^ due to its low density and good thermal/chemical stability.^10^

2D-hBN has a wide band gap (*ca.* 5.2–5.8 eV),^11,12^ which means it is classified as an electrical insulator,^12^ thus it is widely applied as a charge leakage barrier-layer in electronic equipment.^5,13^ Interestingly, 2D-hBN has also been used to tailor the band gap of graphene (creating graphene-hBN interfaces).^13,14^ It has been demonstrated that 2D-hBN's band gap can be decreased/modified by creating thin strips of single layered 2D-hBN nanosheets; thus producing nanoribbons (NRs), which contain a honeycomb lattice with either armchair or zig-zag edges that possess active dangling bonds.^15^ The electronic properties of such nanoribbons are strongly affected by edge termination structures, reconstructions and functionalization.^16^ Moreover, Golberg *et al.*^17^ reported that 2D-hBN-NRs become semiconductors due to doping-like conducting edge states and vacancy defects. Furthermore, by controlling the hydrogenation ratio, the electronic and magnetic properties of zig-zag-terminated 2D-hBN-NRs can be precisely tailored, modulating their band gap.^18^ However, most recently in electrochemistry, 2D-hBN has been computationally explored as a potential electrocatalyst towards the Oxygen Reduction Reaction (ORR) (when computationally supported upon Co, Ni or Cu substrates), where it was shown that the underlying metal support highly influences the electrochemical behaviour of the 2D-hBN.^19^ In other work, Uosaki *et al.* reported 2D-hBN powder immobilised upon gold electrodes showing activity towards the ORR,^20^ proving previous theoretical predictions. Khan *et al.* have reported 2D-hBN powder to be sensitive to the substrate roughness^21^ and surfactant content^22^ towards the ORR when drop-casted onto screen-printed electrodes and reported that 2D-hBN edge plane-sites/defects possibly show heterogeneous electron transfer (HET) kinetics.^23^ In terms of electrochemical properties, Li *et al.* recently developed a highly defective nano-flake hBN film, supported on a glassy carbon electrode (hBN/GCE) and applied it towards the detection of ascorbic acid, dopamine and uric acid, exhibiting wide linear ranges, a low limit of detection and outstanding anti-interference ability, making it a potential candidate for sensor devices.^24^

In order to understand the electrochemical properties of 2D-hBN, this paper utilises Chemical Vapour Deposition (CVD) grown 2D-hBN (finally supported upon SiO_2_) in contrast to 2D-hBN in powder form (as used in all of the above studies), allowing full control of the surface morphology; which is studied with physicochemical, computational and electrochemical characterisation. We demonstrate that the electrochemical response of 2D-hBN can be tailored through the introduction of physical linear defects (PLDs) upon the surface of the 2D-hBN. This transforms 2D-hBN from a previously reported electrochemically inert material into one that gives rise to electrochemically useful signatures/activity. Importantly, given that the current accepted model of 2D-hBN is that of it being an insulator/inert material within electrochemical applications, this work shows that its behaviour is more complex than initially reported.

## 2. Results and discussion

### 2.1 Electrochemical and physicochemical characterisation

**Fig. 1:**
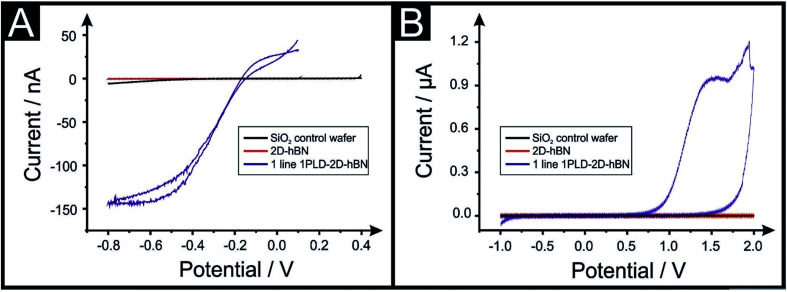
Cyclic voltammetry for 1 mM RuHex/0.1 M KCl (A) and 1 mM Fe^2+/3+^/0.2 M HClO_4_ (B) using a PLD-SiO_2_ wafer as first control (black line), a 2D-hBN electrode (red line) and a PLD-2D-hBN electrode with 1 defective line (blue line) as working electrodes respectively. Scan rate: 50 mV s^−1^; *vs.* Ag/AgCl. Note that the introduction of a PLD gives rise to an electrochemically useful signature.

The electrochemical properties of CVD grown 2D-hBN (supported on SiO_2_) are first characterised with cyclic voltammetry (CV) using Ru(NH_3_)_6_Cl_3_^3+/2+^ (RuHex), which is a near ideal^25^ outer-sphere probe (exclusively sensitive to the electronic structure of the electrode's surface), and additionally (NH_4_)_2_Fe(SO_4_)_2_, (Fe^2+/3+^), which is selected as an inner-sphere redox probe due to its sensitivity to oxide groups on the electrode's surface.^25^Fig. 1 shows that the 2D-hBN electrodes exhibit *no observable* electrochemical activity towards RuHex and Fe^2+/3+^. These results confirm the literature assertion that 2D-hBN is indeed an inert/insulating material.

Our previous work on 2D materials has asserted that active edge plane-sites/defects are the origin of electron transfer,^26^ consequently attention was turned to attempting to introduce such sites, through investigating the creation of PLDs upon the surface of the 2D-hBN (now termed: PLD-2D-hBN). In order to explore this, we physically created, with a diamond knife, a *ca.* 1 mm (length) by 60 μm (width) PLD on the surface of the 2D-hBN electrodes (as described within the ESI†). The PLD-2D-hBN sample was then applied towards the electrochemical detection of RuHex and Fe^2+/3^ (as depicted in Fig. 1). Fig. 1A shows the absence of any observable voltammetric peaks for RuHex when a 2D-hBN electrode was utilised. For controls, a PLD-SiO_2_ wafer was utilised (no 2D-hBN) and an *unmodified* SiO_2_ wafer (no 2D-hBN), which also yielded no electrochemically measurable/observable responses. However, in the case of the PLD-2D-hBN, there is clearly an electrochemical response evident, which is associated to the electrochemical reduction and oxidation of RuHex. It is interesting to note that the electrochemical signature (*viz*Fig. 1A) of the PLD-2D-hBN is sigmoidal in nature, which corresponds to the response expected for a nano-band electrode. The PLD is fabricated with a 1 mm length and 60 micron width, creating a morphology that has no material in the middle, such that the ‘edges’ are 2 × 1 mm lengths and 2 × 60 μm in width. Given that only the edges are active and due to diffusional interaction, the overall response becomes that of a nano-band. This is consistent with the magnitude of the current observed in Fig. 1. Cyclic voltammograms (CVs) shown in Fig. 1B relate to that of the Fe^2+/3+^ probe, which again exhibit no measurable electrochemical activity at the control PLD-SiO_2_ and SiO_2_ wafers, however *again*, for the case of the PLD-2D-hBN there is clearly an electrochemical oxidation peak associated with the redox probe. In summary, it is clear that, with the introduction of a PLD upon the 2D-hBN film/electrode, an electrochemical signal/output is observed which is not present with a pristine 2D-hBN working electrode (and controls). Note; we infer that the PLDs are acting as electroactive sites. CVs of RuHex and Fe^2+/3^ using a PLD-SiO_2_ wafer (*i.e.* a substrate control with no 2D-hBN) are depicted in Fig. S1A and B† respectively, showing the absence of a voltammetric profile when no 2D-hBN is present. Also, the lack of diamond contamination is shown from observing the Raman profile of both SiO_2_ and PLD-2D-hBN (shown in Fig. S1E and F†); thus we are confident the PLDs on the 2D-hBN surface are the origin of the observed electron transfer.

In the above approach we have fabricated a nano-band electrode by introducing PLDs upon a 2D-hBN electrode. Nano-band electrodes are electrochemically characterised by fast mass transport, a high signal to noise ratio, and rapidly achieving steady state; giving rise to enhancements in electroanalytical sensing and kinetic measurements. However, due to the size of fabricated nano-bands upon the surface of the 2D-hBN (*i.e.* nano), the current output is nano-amperes and can be plagued with noise. To overcome this, an array of multiple nano-bands wired in parallel can offer the same enhanced sensitivity of a single micro-band, however with the advantage of a higher total current output. Thus, we sought to introduce multiple PLDs onto the 2D-hBN electrode surface. We explored the response of 1 PLD through to a total of 6 PLDs using RuHex. It was found that additional PLDs created on the surface result in a further improvement in the electrochemical response, but the current does not readily scale with the increasing number of PLDs. This is due to the presence of diffusional interactions between the PLDs as a result of the physical constraints upon the electrode surface. Nevertheless, this demonstrates that the current can be increased from the nano-ampere region to that in the micro-ampere range. Following this we attempted to undertake a voltammetric scan rate study, that is, we change the scan rate from slow (15 mV s^−1^) to fast (250 mV s^−1^) to provide further electrochemical insights. We observed that the voltammetric current diminished upon repeated scan rate studies. In order to explore why this was the case, we performed a full physicochemical and computational characterisation.

**Fig. 2:**
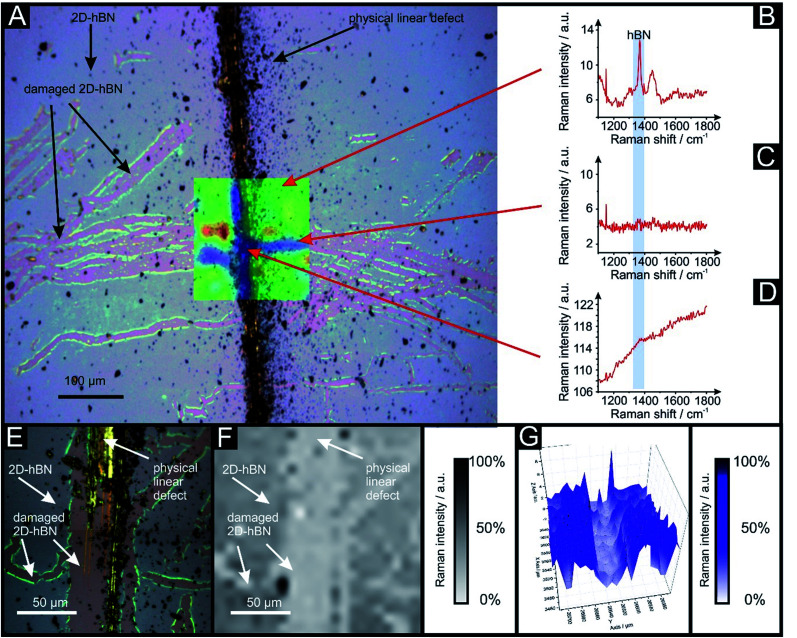
Optical image of PLD-2D-hBN electrode used for a voltammetric scan rate study (28 repeated scans) of 1 mM RuHex/0.1 M KCl, decorated with 6 PLDs resulting in a tree-shape defect pattern. This is due to its electrochemical application. (A) Shows the optical image overlapped with Raman mapping. (B) Shows typical Raman peak of 2D-hBN (1365 cm^−1^), (C) shows Raman peak of a damaged area caused by the HER scanning and (D) shows the Raman peak of a physical linear defect. Higher resolution zoom in area with optical image (E), 2D Raman mapping (F) and 3D Raman mapping (G) of another area of the same PLD-2D-hBN electrode.

**Fig. 3:**
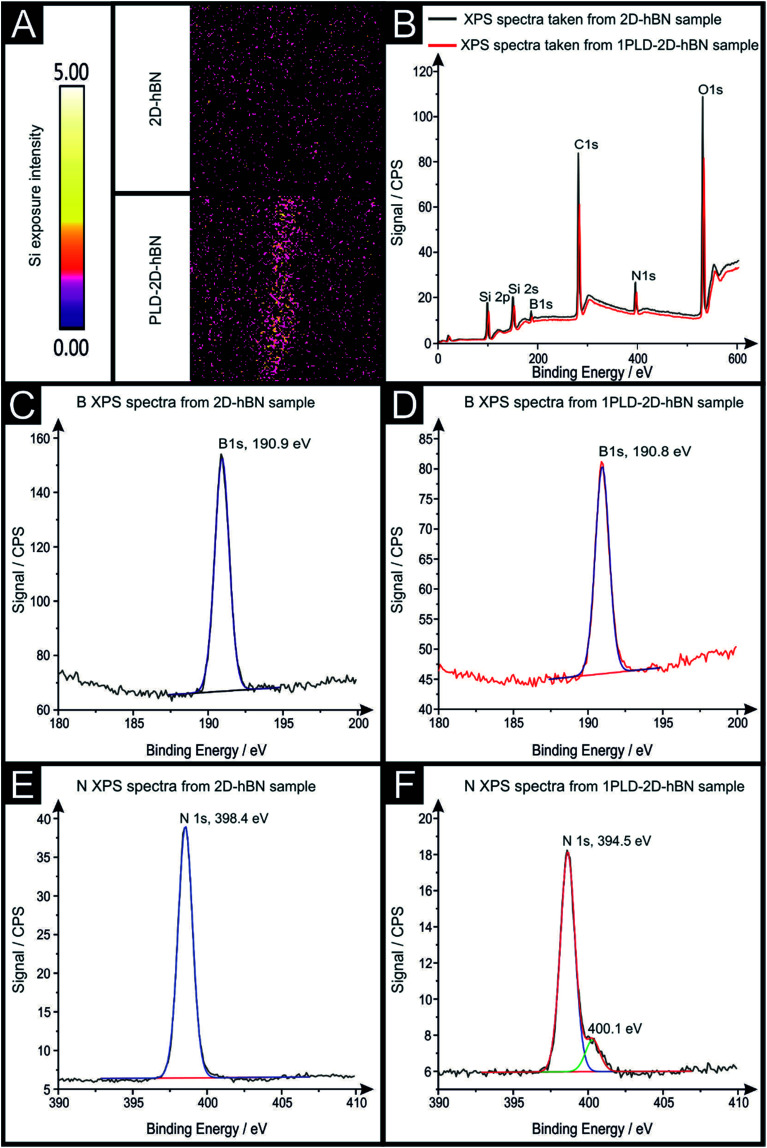
XPS analysis of 2D-hBN and PLD-2D-hBN's edge of a PLD. XPS map of 2D-hBN and PLD-2D-hBN electrode (A), XPS spectra for both locations (B), high resolution XPS analysis of the B 1s components of an 2D-hBN (C), PLD-2D-hBN (D) electrode and high resolution XPS analysis of the N 1s components of an 2D-hBN (E) and PLD-2D-hBN (F) electrode.

Raman mapping characterisation was performed as depicted in Fig. 2, where it can be observed that cracks/defects have formed across the surface from the PLD upon the 2D-hBN surface. Fig. 2 shows damage on the surface of the 2D-hBN caused during electrochemical perturbation (*i.e.* CV scans), with some areas of the electrode resulting in less 2D-hBN coverage. To explore the elemental change upon the surface of the 2D-hBN, X-ray photoelectron spectroscopy (XPS) was performed, confirming the commercially sourced sample to be 2D-hBN. Fig. 3A depicts an XPS map of both unmodified and PLD-2D-hBN samples with the coverage of Si highlighted. It is clear that the PLD has penetrated the 2D-hBN sheet and exposed the underlying Si wafer (PLD-Si wafer was confirmed in Fig. 1 not to be electrochemically active towards RuHex and Fe^2+/3+^). Spot XPS analysis was next performed on the two 2D-hBN sheets and as shown in Fig. 3B, the first sample was an undamaged basal plane (2D-hBN) and the second was at the edge of the induced defect of a PLD-2D-hBN sample. XPS spectra for both locations are shown, B 1s in Fig. 3C and D for unmodified and PLD-2D-hBN samples respectively, and N 1s in Fig. 3E and F for unmodified and PLD-2D-hBN samples respectively. B 1s and N 1s components are seen at *ca.* 190.8 eV and 398.5 eV respectively, with roughly a 1 : 1 stoichiometry. The observed binding energy of these photoelectron peaks strongly correlate to those expected for 2D-hBN.^27,28^ There is a significant amount of carbon and oxygen present on the surface at both sample sites, due to typical adventitious carbon contamination^29^ and from the manufacturing process. During synthesis of the 2D sheet, it is commonly transferred onto the desired substrate using a PMMA polymer^30^ (which has been previously reported to affect physical and electrical properties of CVD grown graphene transferred samples^31^). High resolution XPS analysis of the B 1s and N 1s components at the basal and edge sites are reported in Fig. 3(C), (D), (E) and (F), respectively. There is no observed alteration in the B component, however interestingly a minor peak/signal is observed at 400.3 eV for the edge site N 1s component, which is not observed at the basal site. This additional minor peak at the edge site may be related to protonated amine groups,^27,32^ which represents the hydrogenation of the nitrogen at these edge sites. The O 1s peaks in Fig. S3(A) and (B)† both show O 1s at *ca.* 533 eV, that can be ascribed to surface hydroxyl species or adsorbed oxygen.^33^

### 2.2 DFT computational characterisation

Density Functional Theory (DFT) was used in order to explain the observed beneficial electrochemical response when PLDs are introduced onto the 2D-hBN electrode surface.

#### 2.2.1. Percent of hydrogenation

According to the literature, an increase in the hydrogenation ratio of the edge-plane atoms can turn 2D-hBN from an insulator to a semiconductor, having a noticeable enhancement on the electrical conductivity of 2D-hBN-NRs.^18^ As our PLDs are effectively nano-bands akin to NRs, we perform Density Functional Theory (DFT) calculations in an attempt to find an interpretation for the increased rate of electron transfer and electrical conductivity in the modified 2D-hBN nanosheets; DFT optimisations are fully described in the ESI.†

In order to explore and explain the observed electrochemical signal outputs observed from the PLD-2D-hBN we apply DFT to 2D-hBN-NRs.^15^ We postulate that the PLD introduces hydrogen atoms in the nanoribbons edge site. In order to investigate the influence of varying the hydrogenation ratio of 2D-hBN-NRs on their electrical and electronic properties mono- (mh-hBN-NR) and fully hydrogenated (fh-hBN-NR) edge-planes were simulated in the computational study as shown in Table 1. DFT calculations were explored to investigate the highest occupied molecular orbital (HOMO), the lowest unoccupied molecular orbital (LUMO) and the total density of states (TDOS) with mh-hBN-NRs and fh-hBN-NRs edge planes as well as oxygen-passivated structures (investigated due to the presence of oxygen peaks in the XPS results). Table 1 shows that the energy gap involved in the process of electron transfer decreases significantly by adding hydrogen atoms to the nanoribbon structure, with a change from 6.11 to 3.35 eV at mono-hydrogenated and fully hydrogenated hBN-NRs respectively.

**Table 1:** Electronic clouds delocalization on frontier molecular orbitals (HOMO and LUMO) and their corresponding energy gaps for mh-hBN-NR, fh-hBN-NR, fh-hBN-NR (O–N) and fh-hBN-NR (O–B). The shown energy values were obtained by using B3LYP/LANL2DZ method of calculation. The iso value is 0.02. The *E*_LUMO_–*E*_HOMO_ corresponds to the energy gap (*i.e.* band gap), which is the amount of energy required to transfer one electron from the highest occupied molecular orbital (HOMO) to the lowest unoccupied molecular orbital (LUMO), can indicate the possibility of electron transfer and allow for comparing this property among different structures. Color code: blue: nitrogen; pink: boron; red: oxygen; white: hydrogen

Nanoribbon	HOMO	LUMO	*E*_HOMO_–*E*_LUMO_/eV
mh-hBN-NR	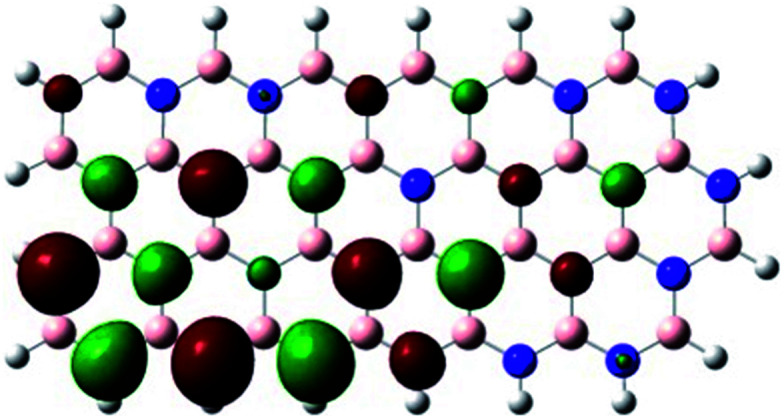	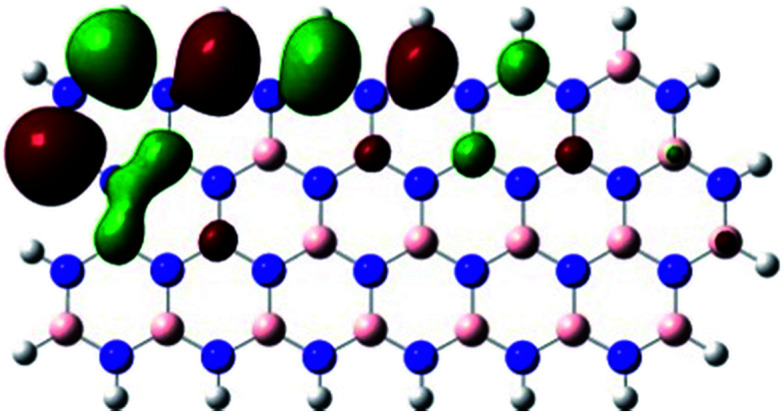	6.11
fh-hBN-NR	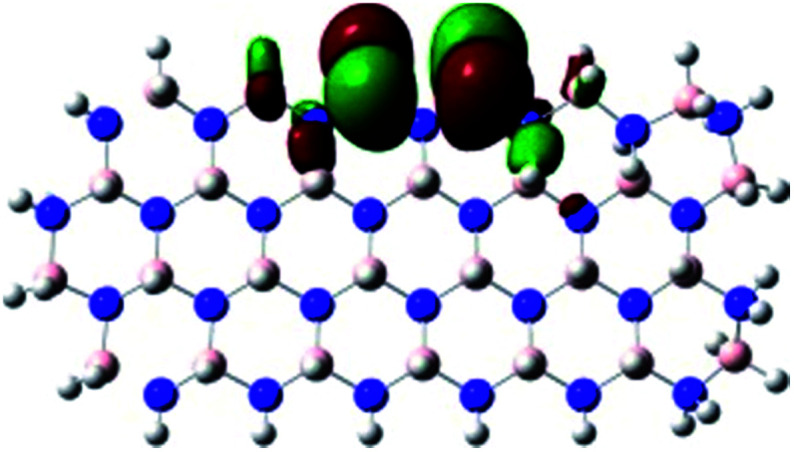	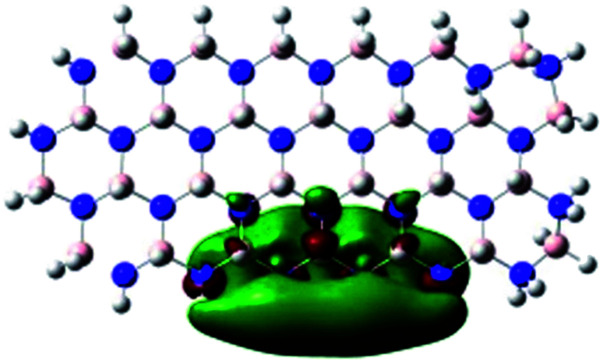	3.35
fh-hBN-NR (O–N)	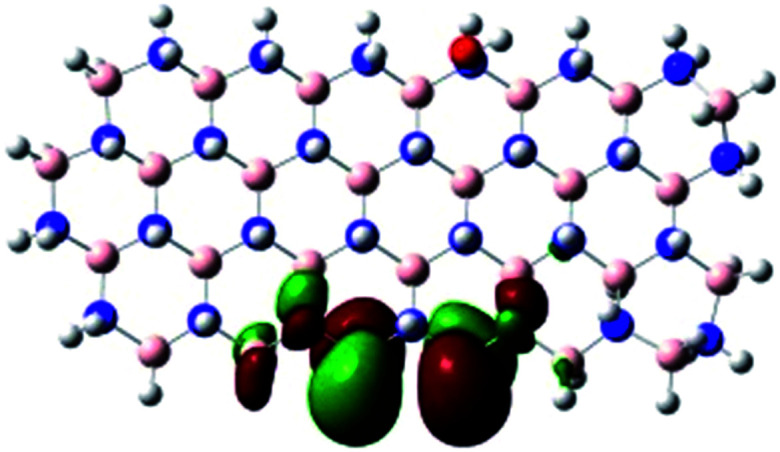	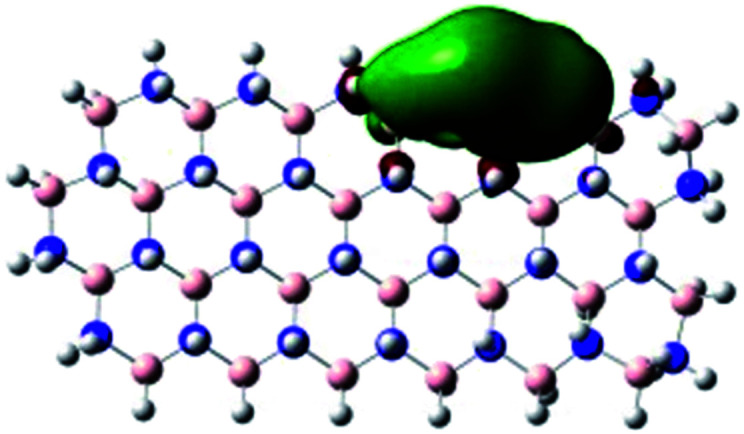	2.84
fh-hBN-NR (O–B)	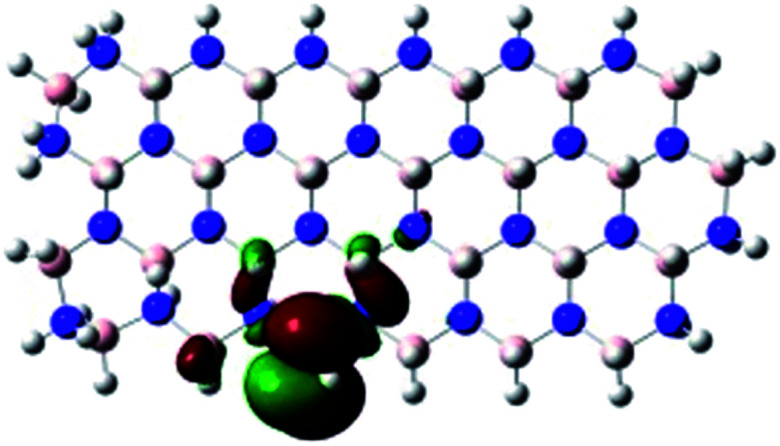	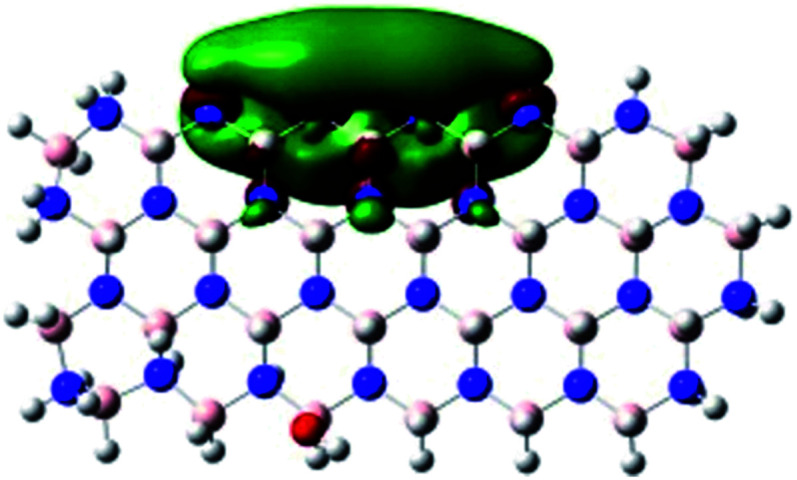	2.36

#### 2.2.2. Molecular orbitals and electron transfer

The electron-transfer process takes place mainly on the edge planes of the 2D-hBN-NRs, with the greatest decrease in band gap being observed on the boron atoms as depicted by the HOMO and LUMO molecular orbitals in Table 1. This is the result of the delocalization of the HOMO molecular orbital on the edge-plane nitrogen atoms; however, the LUMO is delocalized on the edge-plane boron atoms. Interestingly, in fh-hBN-NR (O–N) and fh-hBN-NR (O–B), the LUMO molecular orbitals are delocalized on the oxygen atoms, indicating that oxygen passivation also has a crucial role in enhancing electron-transfer through the 2D-hBN-NR structure. There is a change from 3.35 eV at fh-hBN-NR to 2.84 and 2.36 eV when the oxygen atoms are placed at the nitrogen and the boron atoms respectively.

In summary, the energy gaps are in the order: mh-hBN-NR > fh-hBN-NR > fh-hBN-NR (O–N) > fh-hBN-NR (O–B), where the narrowest energy gap was obtained in the fh-hBN-NR (O–B). Consequently, increasing the number of oxygen-passivated edge-plane boron atoms results in the following transformation: insulator-semiconductor-metallic. Interestingly, the electrical resistivity of 2D-hBN-NR could reach zero in some cases where the degrees of passivation and hydrogenation are at the *maxima*.

#### 2.2.3. Total density of states (TDOS)

**Fig. 4:**
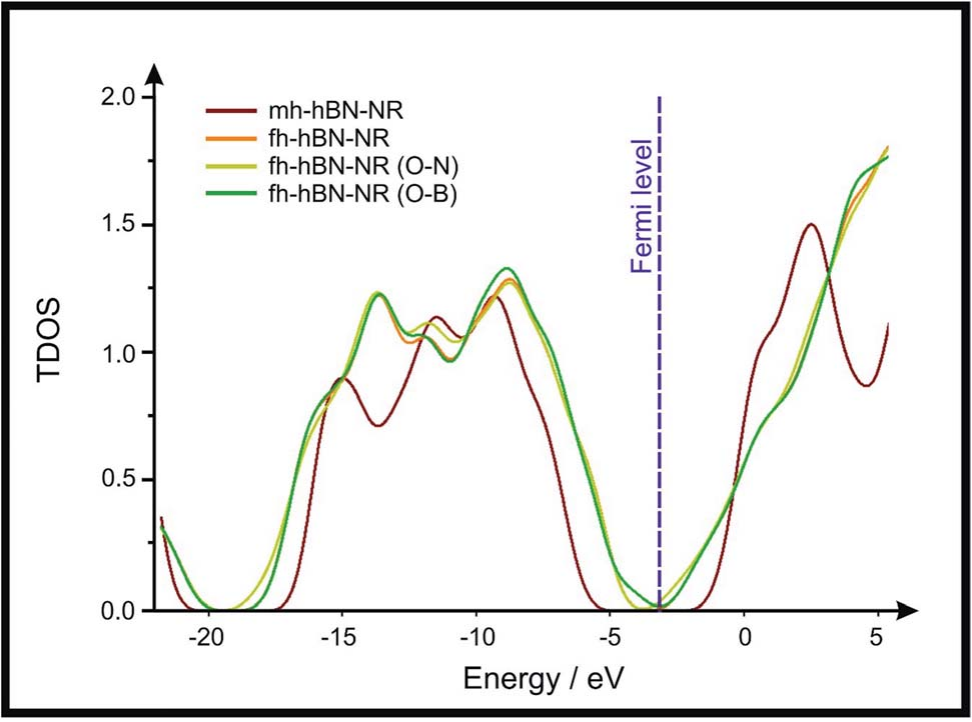
Total density of states (TDOS) of the investigated 2D-hBN nanoribbons (2D-hBN-NRs). The depicted TDOS values were calculated for the above-mentioned structures optimized at the B3LYP/LANL2DZ method of calculation.

In the studied 2D-hBN-NRs, the conduction bands are almost below −5 eV while the valence bands are at 2.5 eV for mh-hBN-NR and at 5 eV for the remaining nanoribbons. It is obvious from the depicted results (Fig. 4) that the band gap in the case of mh-hBN-NR is larger than its counterparts were in fh-hBN-NRs. Moreover, it is notable that some electronic states are added to the conduction band upon oxygen passivation (essentially, increasing the number of oxygen atoms will increase the number of electronic states in the conduction band). This elevation in the conduction band's number of electronic states will increase the electrical conductivity of the nanoribbon, acquiring a semiconductor then a metallic character. Interestingly, passivation of edge-plane boron atoms increases the number of electronic states in the conduction band more significantly than the case where edge-plane nitrogen atoms are passivated instead.

The above confirms the inference that, full hydrogenation and oxygen passivation (presence of O 1s in the XPS results) at the edge-plane boron atoms may be an acceptable interpretation of the enhanced electron transfer and/or electrical conductivity of fh-hBN-NRs with a larger number of edge planes (*i.e.* with induced line defects). In addition, it was found that the total energy of fh-hBN-NR (O–B) and fh-hBN-NR (O–N) (Fig. S2†) are −61 787.2 and −61 784.3 eV respectively. This finding suggests that oxygen atoms attach favourably to edge-plane boron atoms on account of their neighbouring nitrogen atoms. Therefore, it is expected that, when line defects are induced, fh-hBN-NR (O–B) is formed in a larger amount compared to the fh-hBN-NR (O–N) counterpart, which also supports/verifies the observed change in the electrochemical properties.

### 2.3 Electrochemical application

**Fig. 5:**
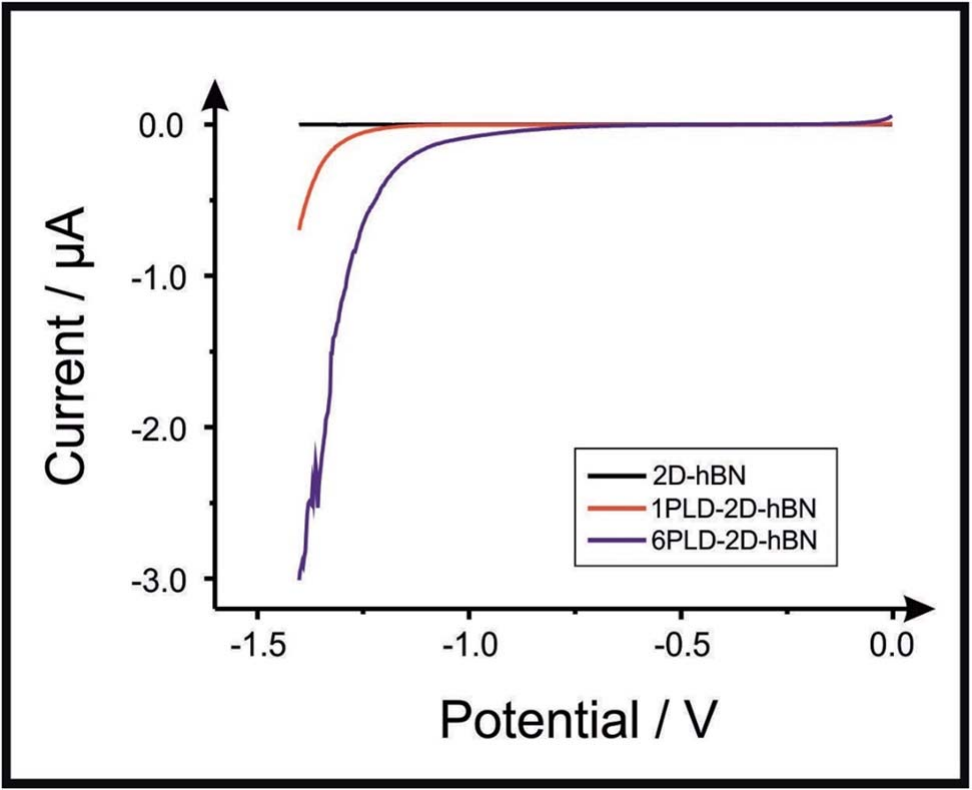
Linear sweep voltammetry of 2D-hBN, 1PLD-2D-hBN and 6PLD-2D-hBN respectively with 0.5 M H_2_SO_4_, depicting the scanning stability experiments towards the HER; showing an increase in the current when more PLD are created (due to an increase in edge plane-sites/defects) when compared to the bare 2D-hBN electrode (scan rate: 5 mV s^−1^; *vs.* Ag/AgCl).

**Fig. 6:**
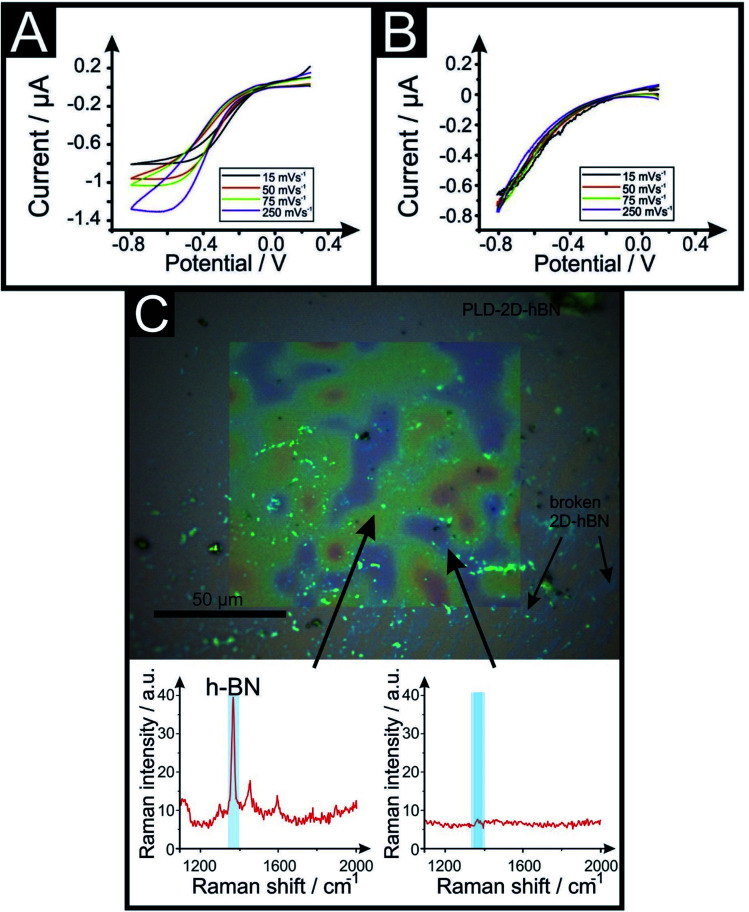
Voltammetric scan rate study of 1 mM RuHex/0.1 M KCl using a 6 line 6PLD-2D-hBN electrode used for 4 (A) and 28 (B) consecutive voltammetric scans to test their electrochemical stability towards multiple sequential potential scanning. Optical image and Raman mapping characterisation of the PLD-2D-hBN after used in (C). Raman spectra is included, showing areas where 2D-hBN is present and its typical Raman peak (red and green colour) and some rips where there is no 2D-hBN peak (blue colour) (scan rate: 50 mV s^−1^*vs.* Ag/AgCl).

2D-hBN has been previously reported as a promising material when incorporated into PEM fuel cells, such as when protecting the membrane from degradation.^34^ The production of molecular hydrogen due to the electrocatalytic reduction of protons (from water) *via* the Hydrogen Evolution Reaction (HER) (2H^+^ + 2e^−^ → H_2_) is a widely studied mechanism and a potential source of sustainable energy supply for the future. With this in mind, in order to test the electrochemical stability of PLD-2D-hBN when applied to electrochemical applications, we explore the HER using a 2D-hBN electrode and a PLD-2D-hBN (with 1 to 6 drawn lines on its surface, as described previously); this is shown in Table S1† and in Fig. 5. Table S1† shows scanning stability experiments of PLD-2D-hBN towards the HER, which is an approach used to benchmark the system against literature reports. As is common practise within the literature, 0.5 M H_2_SO_4_ was used for the HER.^35^Fig. 5 shows the Linear Sweep Voltammogram (LSV) of the 2D-hBN, 1 line 1PLD-2D-hBN and 6 lines 6PLD-2D-hBN respectively, depicting the evolution of the HET kinetics when defects upon the basal plane surface are created (with respect to the HER); leading to an increase in its edge plane-sites/defects when compared to the bare 2D-hBN electrode. It is evident in Table S1† and Fig. 6 that the electrode's performance shifts from a ‘not possible’ (NP) HER when unmodified, to occurring at a HER onset potential of *ca.* −1.19, −1.15, −1.1, −1.08, −0.99 and −0.98 V when 1 to 6 lines are drawn onto the surface, respectively. As we increase the number of PLDs from 1 to 6 there is a corresponding increase in achievable current from −5.23 × 10^−2^ to −6.56 × 10^−1^ μA (recorded at −1.25 V (*vs.* Ag/AgCl) respectively. This is due to the presence of newly generated electroactive sites, *i.e.* more PLDs result in a larger electroactive area.

Fig. 6 compares the electrochemical stability of a 6PLD-2D-hBN electrode used for 4 and 28 RuHex scans respectively at 50 mV s^−1^. As is depicted in Fig. 6A, the 6PLD-2D-hBN electrode used for 4 scans has an increased current and increased electrochemical reversibility than the one used for 28 times (Fig. 6B). Following this use, physicochemical characterisation shows that the passing current clearly disrupts the nature of the film by creating rips and holes, which is confirmed by Raman spectroscopy mapping (shown in Fig. 6C and D) (by the presence/absence of the typical hBN Raman peak at *ca.* 1365 cm^−1^ (red-green areas)^36^).

This confirms a drop in the electrochemical activity upon multiple scanning cycles (28 CV scans), which can be attributed to the damage/destruction of the electrode. This fact is likely due to the dissemination of rips across the surface of the electrode (as previously seen in CVD graphene^37,38^), leading to a lack of conductive pathway, and therefore, to a decrease of its electrochemical performance.

## 3. Conclusions

We have shown, for the first time, that the introduction of PLDs upon the surface of 2D-hBN transforms it from electrochemically inert to exhibiting electrochemically useful signatures. Tailoring the number of PLDs results in a change in the magnitude of the voltammetric current output. A thorough physicochemical (X-ray photoelectron spectroscopy, Raman spectroscopy and scanning electron microscopy), electrochemical (RuHex, Fe^2+/3+^ probes and application towards the hydrogen evolution reaction) and computational (Density Functional Theory) characterisation of the 2D-hBN, both pre- and post-defect creation (*i.e.* PLDs) revealed that the fully hydrogenated amine groups and edge-plane boron atoms passivated with oxygen at the newly created edge plane-sites/defects give rise to recordable/beneficial electrochemical reactivity. Physicochemical and computational characterisation demonstrated that mono hydrogenated 2D-hBN-NRs change from an insulating material with a band gap of 6.11 eV to a semi-conducting material when fully hydrogenated, with a band gap of 2.84 eV for oxygen-passivated boron and 2.36 eV for oxygen-passivated nitrogen. This transition from an insulator to a semiconductor explains the electrochemical observations when using 2D-hBN electrodes for voltammetric studies. It is important for past/present/future studies to note that we observed and documented within this manuscript, that the repeated (potential cycling) use of the 2D-hBN in voltammetric studies creates increasing numbers of defect sites on the 2D-hBN electrode surface, which eventually decrease the electrode's performance; likely due to a decrease in the number of electron pathways. The reduced stability of 2D-hBN in voltammetric studies can introduce doubts into its electrochemical applications, particularly in cases of hybrid 2D materials such as graphene-hBN and other hBN-based nanomaterials, needing further efforts to elucidate the possible contributions of these observations to other 2D materials.

The results described above help to de-convolute the electrochemical properties and responses expected when using 2D-hBN within electrochemical systems. Future studies need to investigate this or other modifications (doping, reversible adsorption, pristine nanoribbons *etc.*), its applicability and implications to real-life macroscopic 2D materials/devices, which will help one take advantage of the unique properties of this modified material and can be applied to a wide range of applications, such as flexible transistors, sensors and optoelectronics to name a few.

## Conflicts of interest

There are no conflicts to declare.

## Supplementary Material

NA-002-C9NA00530G-s001
