# Supplementary material for: Tailoring the electrochemical properties of 2D-hBN via physical linear defects: physicochemical, computational and electrochemical characterisation
Source: Nanoscale Adv. 2019 Nov 21;2(1):264–73. doi: 10.1039/c9na00530g (PMC9418537; doi:10.1039/c9na00530g)
Supplement: NA-002-C9NA00530G-s001 [file NA-002-C9NA00530G-s001.pdf]

Electronic Supplementary Material (ESI) for Nanoscale Advances.
This journal is © The Royal Society of Chemistry 2019

Supporting Information

Tailoring the electrochemical properties of 2D-hBN *via* physical linear defects: physicochemical, computational and electrochemical characterisation

Alejandro García-Miranda Ferrari[1,2], Dale A. C. Brownson[1,2], Ahmed S. Abo Dena[3,4], Christopher W. Foster[1,2], Samuel J. Rowley-Neale[1,2] and Craig E. Banks[1,2*]*

[1]: Faculty of Science and Engineering, Manchester Metropolitan University, Chester Street, Manchester M1 5GD, UK.
[2]: Manchester Fuel Cell Innovation Centre, Manchester Metropolitan University, Chester Street, Manchester M1 5GD, UK.
[3]: Faculty of Oral and Dental Medicine, Future University in Egypt (FUE), New Cairo, Egypt.
[4]: National Organization for Drug Control and Research (NODCAR), P.O. Box 29, Giza, Egypt.

* Correspondence:
(C.E.B.): c.banks@mmu.ac.uk; Tel: ++(0)1612471196

Methods:

All chemicals used were of analytical grade and were used as received from the manufacturer without any further purification. All solutions were prepared with deionised water of resistivity no less than 18.2 MΩ cm and were vigorously degassed prior to electrochemical measurements with high purity, oxygen free nitrogen. The tested solutions were 0.5 M H_2SO_4, 1 mM $(NH_4)_2Fe(SO_4)_2$ ($Fe^{2+/3+}$) in 0.2 M $HClO_4$ and outer-sphere 1 mM $Ru(NH_3)_6Cl_3{}^{3+/2+}$ (Ru/Hex) in 0.1 M KCl.

Electrochemical measurements were carried out using an Autolab PGSTAT204 potentiostat. All measurements were conducted using a three-electrode system with a Pt-wire counter electrode, a silver chloride (Ag/AgCl) reference electrode and a 1 x 1 mm CVD-grown mono-layer 2D-hBN (hBN) on a SiO_2 wafer from Graphene Supermarket completing the circuit. The 2D-hBN samples were in contact with the electrolyte with an area of *ca.* 0.8 x 0.8 cm.

Raman mapping spectroscopic analysis was performed using a Thermo Scientific DXR Raman Microscope fitted with a 532 nm excitation laser at a low power of 6 mW to avoid any heating effects. Spectra were recorded using a 10-seconds exposure time for 10 accumulations in each point. To collect the map we used a step size of 10 × 10 µm, to collect a Raman profile between the region of 1100 and 2000 cm^{-1}. Scanning electron microscope (SEM) images were obtained using a JSM-5600LV (JEOL, Japan) model.

The purposeful modification or deliberate creation of defects upon the mono-layer films consisted of drawing/etching *ca*. 1 mm long-line across the surface of the electrode using a fine diamond scriber from Lattice Gear. Figure S1C and S1D shows SEM images of the modification of PLDs in SiO_2/Si and 2D-hBN on SiO_2 wafer. Figure S1E shows the no-presence of 2D-hBN Raman peak at the newly created edges on a 2D-hBN electrode, and Figure S1F depicts the Raman spectra of the diamond scriber tip (diamond Raman peak[4] at 1332 cm^{-1}), showing that there is no contamination on the samples from it.

The X-ray photoelectron spectroscopy (XPS) data was acquired using an AXIS Supra (Kratos, UK), equipped with an Al X-ray source (1486.6 eV) operating at 300 W in order to perform survey scans and 450 W for narrow scans. All X-rays were mono-chromated using a 500 mm Rowland circle quartz crystal X-ray mirror. The angle between X-ray source and analyser was 54.7°. With an electron energy analyser: 165 mm mean radius hemispherical sector analyser operating in fixed analyser transmission mode, pass energy 160 eV for survey scans and 40 eV narrow scans. A detector with a delay line detector with multichannel plate was utilised.

Density Functional Theory (DFT)

Density Functional Theory (DFT) calculations were performed to unravel the reasons underlying the enhanced electron-transfer properties of 2D-hBN after making line defects and edge plane sites. All DFT computations were performed using B3LYP/LANL2DZ functional implemented in Gaussian 09 package. GaussView Version 05 package[2] was used for visualization of the optimized structures, highest occupied molecular orbital (HOMO) and the lowest unoccupied molecular orbital (LUMO). We used a 6 × 3 (ring × raw) structure of 2D-hBN in order to simulate 2D-hBN nanoribbons (hBN-NR) as shown in Figure S1B. The studied structures of 2D-hBN-NR were either mono- (mh-hBN-NR) or fully hydrogenated (fh-hBN-NR).

The molecular geometry of 2D-hBN-NRs with fully-hydrogenated edge planes (fh-hBN-NRs) were optimized using the B3LYP/LANL2DZ functional and the results were compared with the mono-hydrogenated structure (mh-hBN-NR) (Figure S2). As oxygen presence was detected in the XPS results, oxygen-passivation was also explored, bonding an oxygen atom to either an edge-plane nitrogen or boron atom in order to study both possibilities. It is worth to mention that, in this study the effect of only one atom of oxygen is investigated which means that the obtained results can be maximized by increasing the number of oxygen atoms used for passivating the edge plane nitrogen and/or boron atoms of the nanoribbon. Frequency calculations did not result in any imaginary frequencies indicating the stability of the proposed 2D-hBN-NR structures.

Investigation of the HOMO and LUMO molecular orbitals can give valuable information about the electron-transfer properties through 2D materials such as graphene and 2D-hBN[5]. The so called energy gap (*i.e.* band gap), is the amount of energy required to transfer one electron from the highest occupied molecular orbital (HOMO) to the lowest unoccupied molecular orbital (LUMO), can indicate the possibility of electron transfer and allow for comparing this property among different structures. This parameter is referred to as E_{LUMO}-E_{HOMO}.

The total density of electronic states (TDOS) can give an insight on the electrical properties of materials. Density of states is a concept in solid-state physics, which represents the number of electronic states per unit energy interval[5]. The DOS of a given quantum system/material is an important gadget in order to understand the electrical conductivity and other electronic parameters[5].

Creation of edges:

Figure S1. Cyclic voltammetry of 1 mM RuHex/0.1 M KCl and 1 mM $Fe^{2+/3+}$/0.2 M $HClO_4$ (S1A and S1B respectively) (Scan rate: 50 mVs^{-1}, *vs.* Ag/AgCl) using a SiO_2 wafer (no 2D-hBN) as an electrode. SEM image of a PLD-SiO_2 wafer (S1C) and PLD-2D-hBN electrode (S1D) being utilised towards voltammetric methods. Raman profiles of a PLD-2D-hBN electrode at its newly physical defects (after utilised towards voltammetric) (S1E) and diamond scriber tip with a typical diamond Raman peak[4] at 1332 cm^{-1} (F) showing the lack of 2D-hBN or diamond Raman peak in Figure S1E.

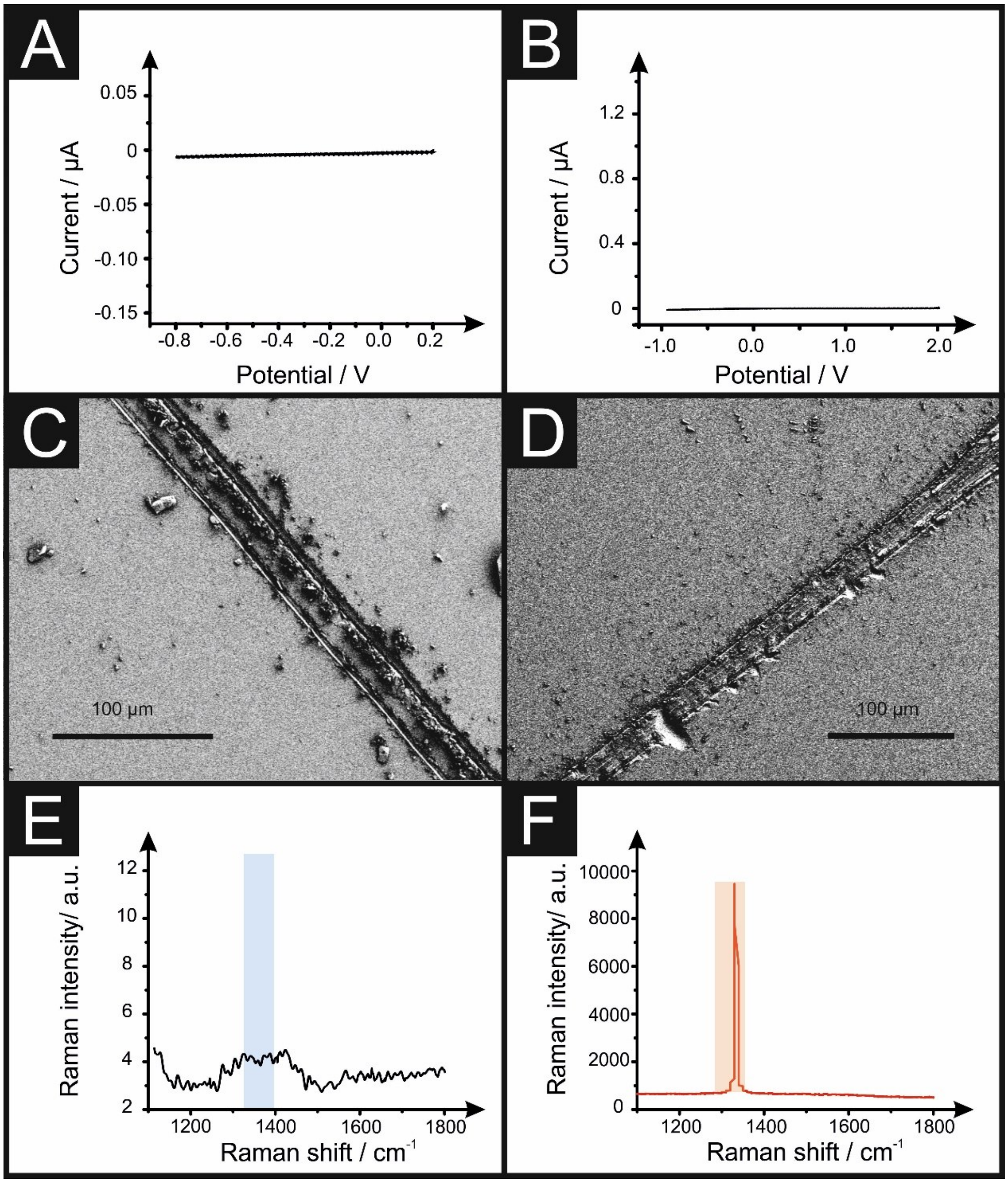

Figure S2. Optimized structures of (A) mh-hBN-NR, (B) fh-hBN-NR, (C) fh-hBN-NR with an edge-plane boron atom passivated with an oxygen atom and (D) fh-hBN-NR with an edge-plane nitrogen atom passivated with an oxygen atom. The shown structures were optimized using B3LYP/LANL2DZ functional. Color code: blue: nitrogen; pink: boron; red: oxygen; white: hydrogen.

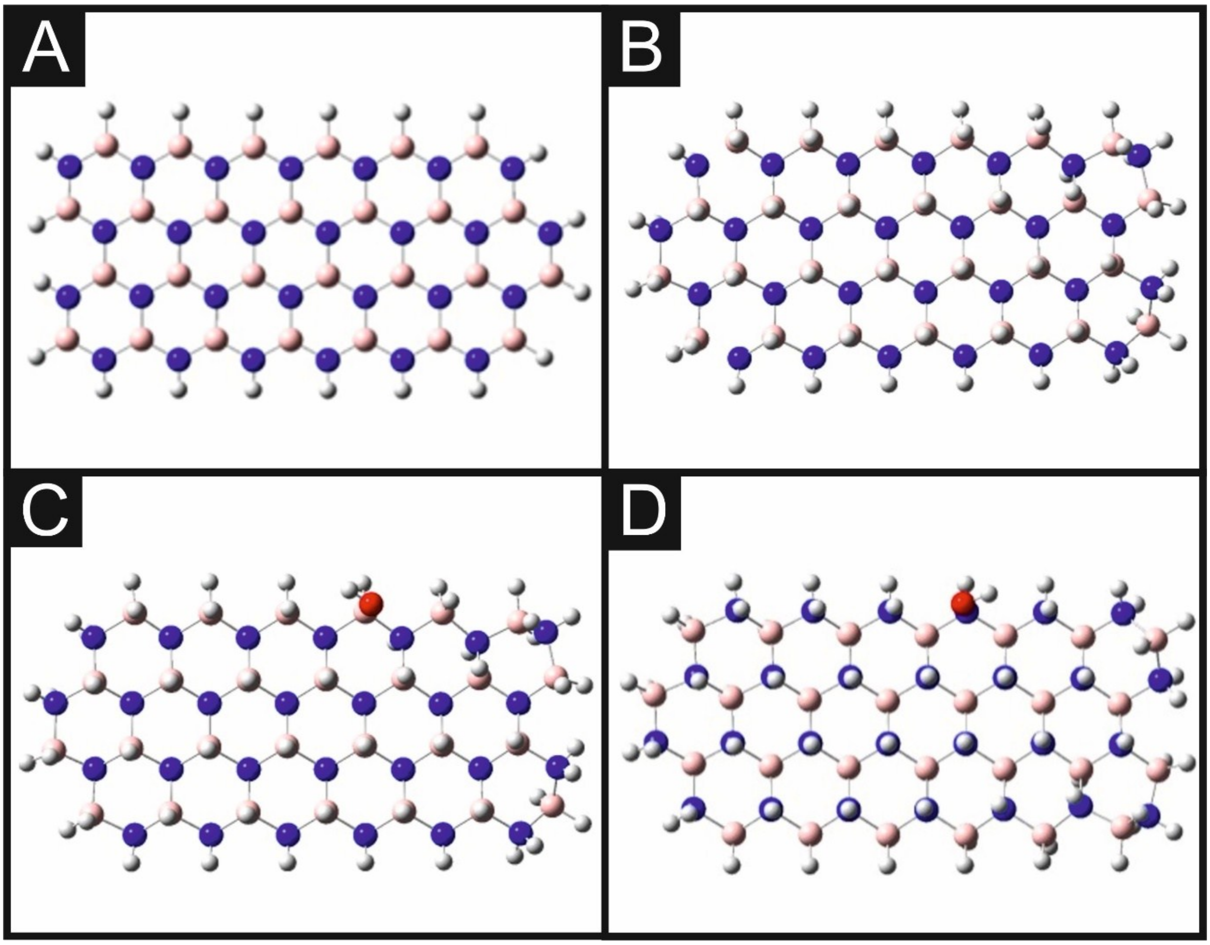

Figure S3. XPS analysis of (A) 2D-hBN and (B) PLD-2D-hBN's edge of a PLD with high resolution XPS spectra for the O1s region.

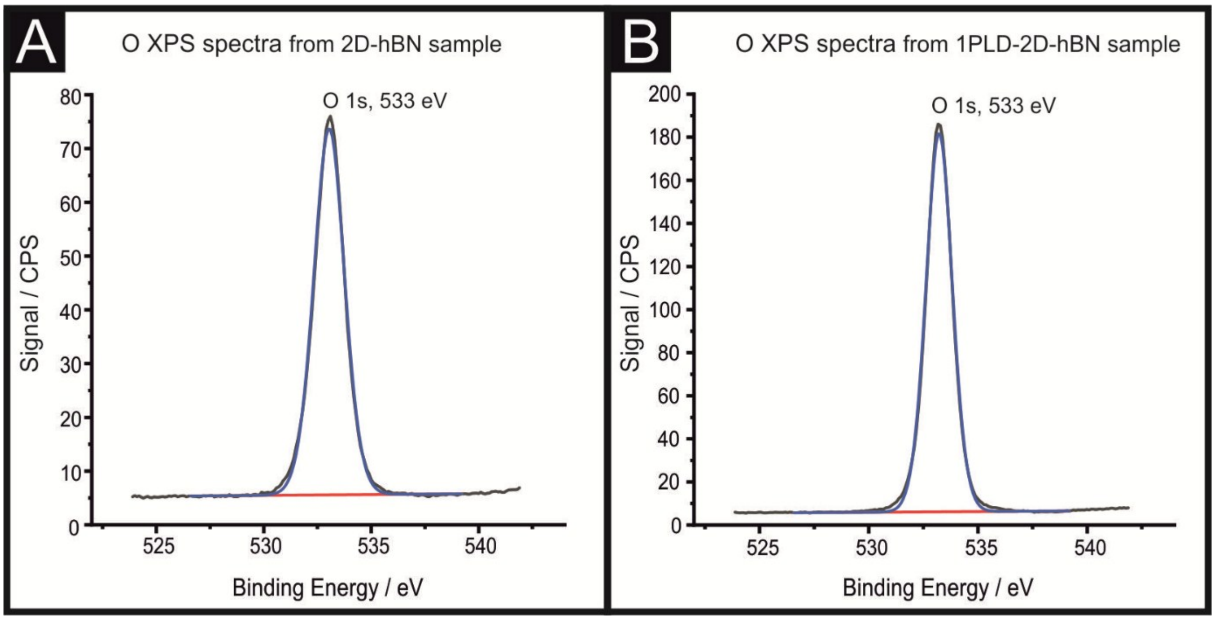

Table 2. Hydrogen Evolution Reaction (HER) analysis for 2D-hBN and 1 to 6 lines PLD-2D-hBN, including HER, onset and current at -1.25 V values. (Scan rate: 5 mV s^{-1}; *vs.* Ag/AgCl). NP; it was not possible to determine a value due to the lack of visible redox peak.

PLDs	***Onset / V***	***Current at -1.25 V / µA***
0	NP	-2.53×10^{-5}
1	-1.19	-5.23×10^{-2}
2	-1.15	-2.14×10^{-1}
3	-1.10	-3.15×10^{-1}
4	-1.08	-4.97×10^{-1}
5	-0.99	-5.03×10^{-1}
6	-0.98	-6.56×10^{-1}

Supporting Information References

[1]. M.J. Frisch, G.W. Trucks, H.B. Schlegel, G.E. Scuseria, M.A. Robb, J.R. Cheeseman, G. Scalmani, V. Barone, B. Mennucci, G.A. Petersson, H. Nakatsuji, M. Caricato, X. Li, H.P. Hratchian, A.F. Izmaylov, J. Bloino, G. Zheng, J.L. Sonnenberg, M. Hada, M. Ehara, K. Toyota, R. Fukuda, J. Hasegawa, M. Ishida, T. Nakajima, Y. Honda, O. Kitao, H. Nakai, T. Vreven, J.A. Montgomery Jr., J.E. Peralta, F. Ogliaro, M. Bearpark, J.J. Heyd, E. Brothers, K.N. Kudin, V.N. Staroverov, R. Kobayashi, J. Normand, K. Raghavachari, A. Rendell, J.C. Burant, S.S. Iyengar, J. Tomasi, M. Cossi, N. Rega, J.M. Millam, M. Klene, J.E. Knox, J.B. Cross, V. Bakken, C. Adamo, J. Jaramillo, R. Gomperts, R.E. Stratmann, O. Yazyev, A.J. Austin, R. Cammi, C. Pomelli, J.W. Ochterski, R.L. Martin, K. Morokuma, V.G. Zakrzewski, G.A. Voth, P. Salvador, J.J. Dannenberg, S. Dapprich, A.D. Daniels, Farkas, J.B. Foresman, J. V Ortiz, J. Cioslowski, D.J. Fox, Gaussian 09W, **2009**.

[2]. R. Dennington, T. Keith, J. Millam, GaussView Version 05, Semichem Inc., Shawnee Mission, KS, **2009**.

[3]. D.-H. Kim, H.-S. Kim, M.W. Song, S. Lee, S.Y. Lee, Nano Converg., 4, **2017**, 13.

[4]. S.A. Solin and A. K. Ramdas, *Phys. Rev. B*, 1, **1970**, 1687-1698.

[5]. A.J. Slate, D.A.C. Brownson, A.S Abo Dena, G.C. Smith, K.A. Whitehead, C.E. Banks, *PCCP,* 20, **2018**, 20010-20022.

[6]. S.J. Rowley-Neale, D.A.C. Brownson, G.C. Smith, D.G.A. Sawtell, P.J. Kelly, C.E. Banks, *Nanoscale* **2015**, 7, 18152-18168.
